# Genotoxicity of Silver Nanoparticles

**DOI:** 10.3390/nano10020251

**Published:** 2020-01-31

**Authors:** Adriana Rodriguez-Garraus, Amaya Azqueta, Ariane Vettorazzi, Adela López de Cerain

**Affiliations:** 1 Department of Pharmacology and Toxicology, Faculty of Pharmacy and Nutrition, Universidad de Navarra, Irunlarrea 1, 31008 Pamplona, Spain; arodriguez.53@alumni.unav.es (A.R.-G.); avettora@unav.es (A.V.); acerain@unav.es (A.L.d.C.); 2 Navarra Institute for Health Research (IdiSNA), 31008 Pamplona, Spain

**Keywords:** silver nanoparticles, genotoxicity, in vitro, in vivo, mouse lymphoma assay, micronucleus test, chromosome aberration test, comet assay

## Abstract

Silver nanoparticles (AgNPs) are widely used in diverse sectors such as medicine, food, cosmetics, household items, textiles and electronics. Given the extent of human exposure to AgNPs, information about the toxicological effects of such products is required to ensure their safety. For this reason, we performed a bibliographic review of the genotoxicity studies carried out with AgNPs over the last six years. A total of 43 articles that used well-established standard assays (i.e., in vitro mouse lymphoma assays, in vitro micronucleus tests, in vitro comet assays, in vivo micronucleus tests, in vivo chromosome aberration tests and in vivo comet assays), were selected. The results showed that AgNPs produce genotoxic effects at all DNA damage levels evaluated, in both in vitro and in vivo assays. However, a higher proportion of positive results was obtained in the in vitro studies. Some authors observed that coating and size had an effect on both in vitro and in vivo results. None of the studies included a complete battery of assays, as recommended by ICH and EFSA guidelines, and few of the authors followed OECD guidelines when performing assays. A complete genotoxicological characterization of AgNPs is required for decision-making.

## 1. Introduction

Nanotechnology has become one of the most sought after technologies in a range of scientific fields and has been identified as a key enabling technology (KET) that provides the basis for further innovation and new products [1]. However, there is currently no standard definition of nanomaterial. Each specific piece of legislation covers only the nanomaterials within its scope; therefore, the definitions of a nanomaterial (NM) and a nanoparticle (NP) can be used only in a defined regulatory context [2]. According to the International Organization for Standardization (ISO), a nanomaterial is a “material with any external dimension in the nanoscale (nano-object) or having internal structure or surface structure in the nanoscale (nanostructured material)” and a nanoparticle is a “nano-object with all external dimensions at the nanoscale, where the length of the longest and the shortest axes of the nano-object do not differ significantly”. Nanoscale is defined as a “length range approximately from 1 nm to 100 nm” (ISO/TS 80004-1:2015) [3]. According to the European Commission, a nanomaterial is “a natural, incidental or manufactured material containing particles, in an unbound state or as an aggregate or as an agglomerate and where, for 50% or more of the particles in the number size distribution, one or more external dimensions is in the size range 1 nm–100 nm” [4]. Although there is no scientific justification for the 1–100 nm threshold, this is based on the fact that many of the particles within the size range, present the specific behavior of NMs [5].

The market offers a wide range of NPs, which vary according to their composition, size, shape, surface materials and other physicochemical characteristics. These materials are normally classified based on morphology, size or chemical characteristics.

NPs can be synthesized through top-down or bottom-up processes. The first method consists of mechanical, physical or chemical processes to reduce bulk materials into NPs; the second, more commonly used method one and the most used, requires molecules, ions or atoms to obtain the nanoparticles [6,7,8]. Depending on their physicochemical properties, NPs are classified as carbon-based, metallic, ceramics, semiconductor, polymeric and lipid-based [8]. 

### 1.1. Silver Nanoparticles Production

According to Nowack et al. [9], it is estimated that some 320 metric tons of silver are produced each year, while Pulit-Prociak et al. [10] have predicted that 800 metric tons of silver nanoparticles (AgNPs) will be produced by 2025. The European Commission estimates that around 20 metric tonnes of AgNPs are marketed each year [5]. 

The sharp increase in the use of nanomaterials in consumer products has led to the creation of several databases to register them. The Consumer Products Inventory (CPI) was created by the Project on Emerging Nanotechnologies (PEN). It contains information about the global market, although it focuses primarily on North America, and lists 443 products as containing AgNPs out of 1827 nanoparticle-containing products [11,12,13].

The European Consumers Organisation (Bureau Européen des Unions de Consommateurs, BEUC) and the European Association for the Co-ordination of Consumer Representation in Standardisation (ANEC) established the 2010 ANEC/BEUC inventory, which contains a specific section on products that contain AgNPs and a list of 141 products [14]. The Nanodatabase is an inventory developed by the Department of Environmental Engineering of the Technical University of Denmark (DTU), the Danish Ecological Council and the Danish Consumer Council. It lists European products that contain nanomaterials and currently includes 3,060 products, 379 of which contain AgNPs [15].

Other sources of information about NPs in the European market are available [5]. Observatory Nano is a database that includes factsheets, briefings and reports and aims to provide an overview of nanomaterials and the areas in which they are used [16]. The Organization for Economic Co-operation and Development (OECD) Database on Research into Safety of Manufactured Nanomaterials provides information on environmental, health-related, and safety-related aspects of nanomaterials [17]. The Nanobiotechnology Laboratory of the Joint Research Centre (JRC) established a repository called Nano Hub, a platform that compiles information for the risk assessment of NPs, such as physicochemical properties and toxicity data [18]. 

### 1.2. AgNPs Characteristics

AgNPs, which are classified as metallic NPs, are some of the most widely used nanomaterials in consumer products; most are smaller than 100 nm and composed of about 20–15,000 silver atoms [19,20]. The most conventional methods to synthesize AgNPs use physical, chemical and mechanical processes, but the biological synthesis of AgNPs using microorganisms, plant extracts or plant biomass is now emerging and appear to be a promising ecological alternative [21,22].

The strong interest in nanomaterials is due to specific physicochemical characteristics that differentiate them from bulk materials made of the same substance. These characteristics include a large surface area, small size and special surface chemistry, and mean that they can undergo surface modifications [9,23,24]. For example, AgNPs constantly release silver ions (Ag+) and at a higher magnitude than silver microparticles of the same weight [9,23]. On the other hand, the stability of NPs may be affected by their elevated surface energy and surface curvature [25,26]. AgNPs may be composed of silver alone or coated with different materials such as synthetic polymers to stabilize them [7,27]. Two of the materials most commonly used to coat and stabilize NPs during manufacturing processes are citrate and polyvinylpyrrolidone (PVP) [13,28,29]. Both compounds lead to a negative surface charge, thereby increasing the stability of NPs through different mechanisms; citrate enhances stability by electrostatic stabilization thereby preventing electrostatic repulsion and aggregation, while PVP improves the stability of AgNPs through steric repulsion. These coatings directly impact the properties of NPs [30,31,32]. Citrate-coated AgNPs have a higher affinity for proteins than PVP-coated AgNPs, and the corona structure they create upon contact with proteins is therefore larger [33].

### 1.3. Uses of AgNPs

Silver has been used for many purposes for thousands of years. Fields of application include jewelry, dental alloys, photography, explosives and because of its antimicrobial properties, drinking water disinfection [9,20]. Indeed, before the introduction of antibiotics, silver had been used to treat open wounds, burns, ulcers and eye infections since ancient times [9,20,34]. Its biocidal function was assumed to be associated with the release of Ag^+^ ions. Silver colloidal suspension, also known as Collargol, has been specifically applied in the field of medicine since 1897 [35]. According to the product patent, Collargol must be a particles dispersion with a colloidal size of less than 25 nm [9,36], which falls within the nanoparticle range (1–100 nm). Collargol can therefore be considered the first AgNP. 

After the discovery of antibiotics, the use of AgNPs decreased dramatically. However, when nanotechnology emerged, the use of AgNPs began to grow again [23]. The antimicrobial activity of AgNPs has been demonstrated, both alone and combined with antibiotics, against multidrug resistant bacteria [37,38,39]. As a result of their broad- spectrum antibacterial activity, strong permeability, low drug resistance [40,41] and anti-inflammatory properties [42,43], AgNPs are currently applied in a range medical products, including gynecological suppositories, wound dressings, silver-coated catheters, contraceptive devices, orthopedic materials and drug-delivery systems [7,25,38,39,41,44,45,46,47,48]. Moreover, the antiviral effects of AgNPs have recently attracted attention, since they have demonstrated remarkable activity against human immunodeficiency virus (HIV), hepatitis B virus (HBV), herpes simplex virus (HSV), respiratory syncytial virus (RSV), monkeypox virus, tacaribe virus (TCRV), RNA viruses and adenovirus type 3 [44,49]. At present, they are being studied as a potential alternative to treat human infectious diseases, especially influenza virus infections in which antivirals have generally proven to be unsuccessful [50]. Moreover, AgNPs have shown promising antitumor effects [44]. 

Due to their optical properties and high electrical and thermal conductivity, AgNPs are also used in biosensors, photothermal therapy and medical diagnosis to detect antibodies [13,44,51]. AgNPs in colloid form are used in cosmetics because of their antimicrobial and antifungal properties and in toothpastes and skin care products, with a maximum concentration of 1% [13,52]. Bath and shower products, soaps, shampoos, anti-perspirants, facial and eye care cosmetics, and facial cleansers all may contain AgNPs [52]. 

AgNPs are used in the food industry to inhibit the microorganisms that cause food poisoning and spoilage and in dietary supplements and food contact materials. They are also incorporated into filters to purify drinking water, as they are effective against multidrug-resistant bacteria [7,23,39,41,42,46,52,53,54].

Furthermore, AgNPs are included in household items, textiles fabrics [13,47,53] and swimming pool filters [7,39,41,42,46,52,53]. Due to their optical, electrical and thermal properties, they are used in electronics, imaging, catalysis and biosensing, and in products such as washing machines and refrigerators [13,51,55]. The morphology—dependent properties of noble metal nanoparticles, with a focus on localized surface plasmon resonance and local field enhancement, mean that the AgNPs are applied in fields such as Raman spectroscopy, fluorescence enhancement, analytics and sensing [25].

### 1.4. Genotoxicity Evaluation

The use of AgNPs in a wide range of products is rising and, as a result, interest in their biosafety is growing. Human exposure to NPs occurs during different phases of the life cycle of a consumer product: initial synthesis, production, manufacturing, industrial emissions, product degradation, disposal and food chain contaminations [13,56]. Humans are directly exposed to AgNPs via several routes, including inhalation, dermal contact and oral ingestion, which is the most significant route. According to Boudreau et al. [57], the human dietary intake of silver generally varies from 0.4 to 27 μg a day; however, Vila et al. [53] and Wijnhoven et al. [46] claim that the dietary intake of AgNPs is between 70 to 90 μg a day, and, Li Tang and Xue [29], agree that the daily intake of silver in humans may be as high as 90 µg.

In light of the rapidly growing use of AgNPs, a testing strategy is required. Since 2004, the Scientific Committee on Emerging and Newly Identified Health Risks (SCENIHR), the Scientific Committee on Consumer Safety (SCCS), the European Food Safety Authority (EFSA) and the European Medicines Agency (EMA) have been working on assessing the risks of nanomaterials [1]. Genotoxicity is a key element of this safety assessment. It is essential to identify potential mutagens and/or human carcinogens through the detection of primary DNA lesions, gene mutations, chromosomal damage or recombination.

**Figure 1:**
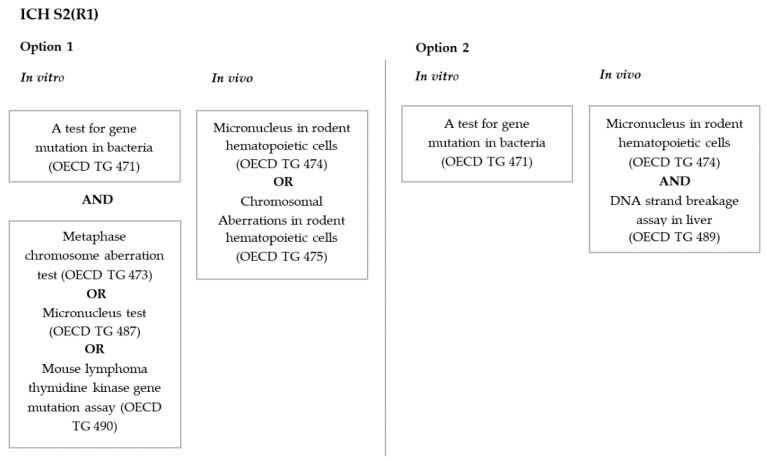
Scheme of the ICH S2(R1) strategy for a genotoxicity assessment.

Guidance issued by the International Council for Harmonization of Technical Requirements for Pharmaceuticals for Human Use (ICH) on genotoxicity testing and data interpretation for pharmaceuticals intended for human use (ICH S2(R1)) [58] proposes two different options for evaluating the genotoxicity of new pharmaceuticals (Figure 1). This guidance focuses on new “small molecule” drug substances and makes no specific reference to NPs. Both testing strategies start with a test for gene mutation in bacteria (Figure 1), which is not applicable to NPs because bacteria cannot internalize NPs [32,41,59]. In addition, given that AgNPs are antimicrobial agents, the experimental system (i.e., bacteria) is incompatible with the product to be tested (AgNPs). For those reasons, it has been concluded that the gene mutation test in bacteria is ineffective for assessing the genotoxicity of nanomaterials [60]. Therefore, as explained later, organizations such as EFSA and ISO recommend that the gene mutation assay in bacteria be replaced by assays that use mammalian cells.

ISO states that metallic nano-objects present great concern, since they are highly reactive, and as a result, published the document “ Biological evaluation of medical devices: Part 22, Guidance on nanomaterials” in 2017 [3]. For the genotoxicity testing of nanomaterials, it recommends the use of mammalian cell systems instead of bacteria due to the capacity of the former to internalize the particles. The in vitro assays that have commonly been used to assess the in vitro genotoxicity of nanomaterials according to the ISO technical report are as follow: the mammalian cell gene mutation tests using the hypoxanthine-guanine phosphoribosyl transferase (HPRT) or the thymidine kinase (TK) gene, the micronucleus (MN) test and the comet assay (neutral, alkaline or enzyme-modified assay). The most commonly used in vivo tests are the MN test in rodent blood or bone marrow erythrocytes, chromosome analysis in rodent bone marrow cells and the alkaline comet assay in the target tissue/s, although the choice of assays should be made on a case-by-case basis and requires justification and documentation. 

**Figure 2:**
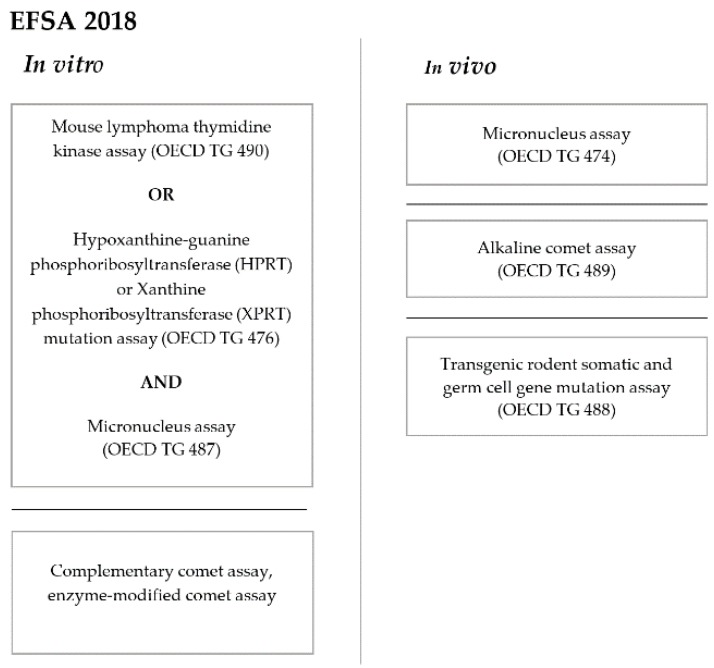
Scheme of the EFSA 2018 strategy for a genotoxicity assessment of NPs.

On 29 May 2018, the EFSA published “Guidance on risk assessment of the application of nanoscience and nanotechnologies in the food and feed chain: Part 1, human and animal health” [61], a set of guidelines that proposed of a strategy for assessing the genotoxicity of NPs (Figure 2). Each assay should be performed in accordance with the corresponding OECD guideline recommendations (as in the case of the ICH). To study gene mutations, both the in vitro mammalian cell gene mutation tests that use the HPRT and XPRTT genes (OECD TG 476) [62] or the thymidine kinase (TK) gene (OECD TG 490) [63] are suitable. To study structural and numerical chromosome aberrations (CA), the in vitro mammalian cell micronucleus (MN) test (OECD TG 487) [64] should be performed. According to the EFSA (2018), the in vitro comet assay and, more specifically, the in vitro modified comet assay for the detection of oxidative DNA lesions, may provide complementary information of the materials that produce oxidative stress, such as AgNPs. With respect to in vivo testing, an in vivo micronucleus test (OECD TG 474) [65] and/or an in vivo mammalian alkaline comet assay (OECD TG 489) [66] and/or a transgenic rodent somatic and germ cell mutation assay (OECD TG 488) [67], should be carried out if one of the in vitro tests indicates genotoxicity or if any of the in vitro assays is not suitable for evaluating NPs [61]. The specific strategy for a given nanomaterial should be decided on a case-by-case basis on the information available and expert opinions.

Both the ISO/TR 10993-22:2017(E) and EFSA 2018 guidelines consider it essential to identify relevant properties in terms of biological and toxicological aspects and to understand the behavior of a nanomaterial through physicochemical characterization that includes the chemical composition, physical description and extrinsic properties of the material [3,61]. Due to knowledge gaps, there is no single consensus list to adequately describe a nanomaterial, but both guidelines suggest some parameters considered important by experts [3,61]. Not all parameters may apply to each specific material, and additional properties should be considered on a case-by-case basis, but each characterization must be justified. The list in the EFSA guideline is more extensive, but both recommend that the following properties be determined: composition, purity, particle size, agglomeration, aggregation, particle shape, surface chemistry, surface charge, surface area, solubility and dispersibility of the nanomaterial [3,61]. Both guidelines provide information about the different techniques to be used on each determination and the relevant ISO guidelines for each of the methodologies. Techniques such as dynamic light scattering (DLS), electrophoretic light scattering (ELS), scanning or transmission electron microscopy (SEM,TEM), single particle ICP-MS, x-ray diffraction (XRD) and spectroscopy are generally used to characterize NPs [3]. 

In summary, although several strategies for testing the genotoxicity of NPs are available, the risk assessment of NMs should be performed on a case-by-case basis, based on pertinent information [2]. Moreover, it is important to take the physicochemical characteristics of the nanomaterial into account to correctly interpret the results [3,58,61,68]. 

## 2. Objective

In view of the extent of human exposure to AgNPs and the importance of genotoxicity testing, information on the genotoxic effects of these products is required to ensure that they are safe to use. For this reason, a bibliographic review of the genotoxicity of AgNPs was carried out. 

## 3. Search Strategy

A bibliographic search was carried out through the PubMed database [69] by entering key words in the advanced search builder. These key words were: genotoxicity OR mutagenicity AND silver AND nanoparticles (included in the title/abstract). In this first phase of the selection process, 146 articles were retrieved through the database search. The most recent reviews of relevance regarding AgNPs genotoxicity dated from 2012–2013; therefore, a filter was applied to select articles published in the last 6 years. This resulted in 106 articles to be assessed for eligibility.

In the first phase the articles were selected by reading the abstract and applying the following inclusion and exclusion criteria: 

Inclusion criteria:

- The article contains information on in vitro or in vivo genotoxicity testing of AgNPs.
- The genotoxicity assay(s) included in the article is (are) well established and validated genotoxicity assays (i.e., those included in the EFSA, ISO or ICH guidelines on genotoxicity testing).

If the answer to both criteria was “yes”, the article was selected.

- Exclusion criteria: The article deals with environmental and ecotoxicity studies
- The article deals with gene expression evaluations.

If the answer to either exclusion criteria was “yes”, the article was rejected.

In case of doubt the article was selected for in depth analysis in the second phase; this involved reading the full article. After applying the inclusion and exclusion criteria, 41 articles were selected. 

Seventeen reviews were retrieved during the bibliographic search; all articles referenced in these reviews were analyzed and included in the tables if they had not already been included and fulfilled the inclusion criteria. This led to the inclusion of two further articles in the selection. 

As mentioned above, only articles that referred to well-established genotoxicity assays were selected. The following in vitro assays the were considered: (1) the in vitro mammalian cell gene mutation tests using the thymidine kinase gene (MLA) (OECD TG 490) [63] (or the HPRT and XPRT genes (OECD TG 476) [62]; (2) the in vitro mammalian cell MN test (OECD TG 487) [64]; (3) the in vitro mammalian chromosome aberration test (OECD TG 473) [70]; and (4) the in vitro comet assay (both standard and modified with oxoguanine glycosylase 1 (OGG-1), formamidopyrimidine DNA glycosylase (Fpg) and endonuclease III (Endo-III)). The following in vivo assays were included: (1) the mammalian erythrocyte MN test (OECD TG 474) [65]; (2) the in vivo mammalian alkaline comet assay (OECD TG 489) [66]; and (3) the mammalian bone marrow chromosome aberration test (OECD TG 475) [71]. With respect to articles on AgNPs of different sizes, only information on NPs around 100 nm in size was extracted.

The results of the review were summarized in six tables and data from each in vitro or in vivo assay were included in each one. The tables were arranged in logical order of genotoxicity assessments, i.e., in vitro assays followed by in vivo assays. Table 1 contains the in vitro MLA results, Table 2 the in vitro MN results, Table 3 the in vitro comet results (both standard and enzyme-modified), Table 4 the in vivo MN results, Table 5 the in vivo chromosome aberration results, and Table 6 the in vivo comet results (both standard and enzyme-modified). Evaluations of each table were organized according to NP size, from smallest to largest, and the results included were those provided and interpreted by each author. Negative and positive results were recorded in tables without any information about the ‘intensity’ of positive results.

## 4. Results and Discussion

### 4.1. In Vitro Studies

A total of 43 articles were selected for data extraction: 31 were in vitro, 11 were in vivo and one was both in vitro and in vivo. In vitro studies were used in almost all articles retrieved (32/41), with the following distribution: the MLA was used in one article (Table 1), the MN assay in 16 articles (Table 2) and the comet assay in 23 articles (Table 3). Although retrieved from the search, two articles that included the comet assay were rejected, due to lack of consistency between the results presented in the figures and the results described. 

With respect to the MLA assay, Guo et al. (2016) [72] treated L5178Y cells (in suspension) with 20, 50 or 100 nm citrate- or PVP-coated AgNPs for four hours. The test was carried out in accordance with OECD TG 490 [63], and the results were evaluated in accordance with the guideline: “Positive responses require a concentration-dependent increase in mutant frequency (MF) and an induced MF after treatment, in one or more cultures, above the global evaluation factor (GEF) of 126 mutants per 10^6^ cells.” As shown in Table 1, the results obtained for every NP size and coating were positive (Table 1). 

The in vitro MN assay was used in 16 of the papers selected, with a total of 59 determinations (i.e., assays performed with different cell lines, treatment durations and concentration ranges); 39 of these presented positive results and 20 presented negative results (Table 2). 

OECD TG 487 [64] was followed in studies carried out by five authors: Sahu et al. [73], Ivask et al. [74], Guo et al. [72], Sahu et al. [75] and Sahu et al. [76]. According to OECD TG 487 [64], human peripheral blood lymphocytes and some rodent cell lines such as CHO, V79 and L5178Y or human cell lines such as TK6 are considered suitable for performing this assay; however, other cell lines such as HT29, Caco-2, HepG2 and A549, which have been used in several studies, have not been extensively validated by OECD TG 487 [64]. 

Many of the studies in this review have used other human cell lines as experimental models for the MN assay, including: human lymphocyte cell lines (JURKAT, WIL2-NS, TPH-1) by Ivask et al. [74] and Butler et al. [77]; human bronchial epithelial cells (HBEC) by Lebedová et al. [78]; human breast cell lines (MCF-10A, MCF-7, MDA-MB-231) by Roszak et al. [52]; and human keratinocytes HaCat by Bastos et al. [31]. Some studies detected differences in the sensitivity of cells [52,72,76,79].

With respect to treatment schedule, the guidelines recommend a short period of 4–6 h with or without metabolic activation, followed by removal of the test chemical and sampling at a time equivalent to about 1.5–2.0 normal cell cycle lengths after the beginning of treatment. Continuous treatment without metabolic activation for 1.5–2.0 cell cycle length is also recommended (OECD TG 487) [64]. In the studies reviewed, treatment usually lasted 24 h or 48 h (40–44) with a few exceptions that lasted 4 h or 72 h [72,76,85]. Almost all studies tested uncoated AgNPs, except for some that used PVP-, citrate- or branched polyetherimide (bPEI)-coated AgNPs [72,74,84]. The size of AgNPs tested varied from 5–100 nm and treatment concentrations ranged from 0.025–400 μg/mL (Table 2).

Regarding evaluation and interpretation of results, providing that all acceptability criteria indicated by the OECD guideline were fulfilled, an in vitro MN test was considered clearly positive when at least one of the concentrations tested exhibited a statistically significant increase in micronuclei compared to the negative control, the response was dose-related and any of the results fell outside the distribution of the historical negative control data (OECD TG 487) [64]. 

These criteria were applied by Sahu et al. [73], Ivask et al. [74], Guo et al. [72], Sahu et al. [75] and Sahu et al. [76]. In the other studies, results were statistically evaluated by comparing data from treated cells to data from negative controls. P values of <0.05 were considered positive.

The in vitro comet assay was used in 23 of the articles selected, with a total of 103 determinations (i.e., assays performed with different cell lines, treatment durations and concentration ranges) (Table 3). 

The different versions were performed and produced positive and negative results; 79 used the standard (ST) version (63+/16−); 15 used the Fpg-modified version (9+/6−); eight used the Endo-III-modified version (6+/2−); and one used the OGG-1-modified version (+). No OECD guidelines exist for the in vitro comet assay but, according to this review, it is the most commonly used in vitro assay to assess genotoxicity of AgNPs. This finding is consistent it with a previous observation that the in vitro comet assay is the most commonly used method for assessing the genotoxicity of NPs [86].

A wide variety of cells were used in the in vitro comet assay. Treatment duration ranged from 4–48 h and concentrations ranging from 0.025–200 μg/mL. Regarding AgNPs characteristics, uncoated AgNPs and those coated with different chemicals, including bPEI, poly(vinyl alcohol) (PVA), sodium bis(2-ethylhexyl)-sulfosuccinate (AOT), cetyltrimethylammonium bromide (CTAB), bovine serum albumin (BSA), poly-L-lysine (PLL), PVP and citrate, have been tested, with sizes ranging from 5–131.5 nm (Table 3). In all studies, the results were statistically evaluated by comparing treated cells to untreated control cells; those with p values of <0.05 were considered positive.

Some of the in vitro studies studied the uptake of AgNPs inside the cells, which is an essential determination to prove that there has been contact between the cell organelles and the AgNPs [53,72,73,77,81,82,83,88,89,91,92,98]. Uptake was demonstrated through several methods such as transmission electronic microscopy (TEM), side scatter (SSC) intensity analysis by flow cytometry, confocal microscopy, Raman spectroscopy and scanning transmission electron microscopy (STEM). When AgNPs are internalized, they are usually stored in vesicles in the cytoplasm. There is no evidence that they the nucleus but there is a theoretical possibility of direct DNA contact when the nuclear membrane breaks down during mitosis [29,31]. Moreover, it is worth noting that AgNPs interactions with the cell membrane can also produce toxic effects, mainly due to oxidative stress. Several authors have studied the influence that the release of Ag+ ions may have on AgNPs genotoxicity; some concluded that that their role is negligible, whereas others obtained inconclusive results or indirect evidence of their influence on the AgNPs cytotoxicity and genotoxicity [74,77,82,83].

There is a critical point during in vitro assays to test AgNPs, especially when cells in suspension are used. After treatment, cells are generally centrifuged to remove the AgNPs, but in many cases, complete separation of solid AgNPs from the cells may not be possible. This is an important factor that can affect results, especially in assays in which the results are not evaluated immediately after treatment but following an incubation period. In the MLA, for example, the treatment period (3–4 h or 24 h), is followed by the subculturing of for 48h and cell seeding for colony growth for 12–14 days; if the NPs have not been completely removed, the treatment period can be extended from 3–4 h or 24 h to as much as 17 days. This is a technical factor that may increase in vitro sensitivity.

The influence of AgNPs size on the genotoxic effect has been addressed by some authors, most of whom found a negative correlation: 5, 10 and 20 nm AgNPs are more cytotoxic and genotoxic than analogous 40, 50, 75 and 100 nm AgNPs [72,76,77,87,91]. Lebedová et al. [78] found no clear evidence of a size-dependent effect between 5 and 50 nm AgNPs and Souza et al. [81] observed a positive correlation; they found that 100 nm AgNPs were more toxic than 10 nm AgNPs. In Table 1, Table 2 and Table 3, the correlation between AgNPs size and genotoxicity is not evident because the results are expressed only as positive (+) or negative (−) and the dose-response relationship is not reflected. In general, positive results have been obtained in the three in vitro assays with AgNPs of different sizes. Lebedová et al. [78] and Roszak et al. [52] obtained negative results in the MN assay with HBEC and several human breast cell lines that were not included in the OECD TG 487 as validated or frequently used cells [64]. 

With respect to the influence of AgNPs coating, no clear correlation was observed across the studies reviewed (Table 1, Table 2 and Table 3). Nevertheless, some authors have studied this aspect and obtained different results with coated and uncoated AgNPs. Ivask et al. [74] found that bPEI-AgNPs produced a higher increase in MN than citrate-AgNPs, and suggested that this was due to the fact that bPEI-AgNPs present stronger cellular adhesion and internalization. Guo et al. [72] observed greater effects in citrate-AgNPs than in PVP-AgNPs, although the effect of coating was less important than the effect of size. Brkic et al. [89] found that intensity of DNA damage measured by comet tail length and intensity, was dependent on AgNPs coating, with PLL-AgNPs and CTAB-AgNPs the most and least damaging, respectively. Finally, Wang et al. [84] observed that uncoated AgNPs caused more DNA damage than PVP-AgNPs in HepG2 cells (comet assay), but the opposite was observed in the MN assay.

### 4.2. In Vivo Studies

*In vivo* studies were included in 12 of the 43 articles retrieved, with a total of 102 determinations (in terms of NPs size, coating, animal model, tissue studied, treatment route and duration and sampling time). Thirty-two of these showed positive results and 69 of them showed negative results. The in vivo MN test was used in seven articles, with a total of 28 determinations (17+/11−) (Table 4). The in vivo CA test was used in three articles, with three determinations (3+) (Table 5). Finally, the in vivo comet test was used in seven articles, with 70 determinations performed with the different versions; 42 ST (6+/36−), 24 Fpg-modified (2+/22−), two Endo-III modified (2+) and two OGG-1 modified (2+) (Table 6).

None of the articles that included the in vivo MN test referred to OECD TG 474 [65]. Mice, rat and rabbit animal models were used, and micronuclei were analyzed in liver, blood or bone marrow cells. AgNPs were administered orally (p.o.) (13/28) or intravenously (i.v.) (15/28), in either single- or repeat-dose studies (Table 4). All single-dose treatments were administered through the i.v. route, whereas repeated-dose treatments were administered through either the i.v. route for 3 days or the p.o. route for 5, 7 or 28 days (Table 4). Animals were given uncoated AgNPs or PVP-, silicon- or citrate-coated AgNPs, arranging in size range from 5−629 nm. Doses were higher for oral treatments (4−250 mg/kg b.w.) than for intravenous treatments (0.5−25 mg/kg b.w.). In all studies data from treated animals were statistically compared to data from untreated animals, and those with p values of <0.05 were considered positive. The results do not appear to be influenced by NPs size (Table 4). With respect to surface functionalization, uncoated and citrate-AgNPs produced positive results, whereas PVP-AgNPs and silicon-AgNPs produced negative results, except in the study by Wang et al. (2019) [84], who observed that both uncoated and PVP-coated AgNPs administered for 28 days were positive, albeit only at the highest dose (Table 4). 

Table 5 shows the results of the in vivo chromosome aberration (CA) assay, which was used in three of the articles selected. None of the articles analyzed followed OECD TG 475 [64]. CAs were analyzed in bone marrow cells of rats treated through the i.v., p.o., or intraperitoneal (i.p.) route for 1, 5 or 28 days, respectively, with uncoated AgNPs measuring 10 nm or 6−629 nm in size. The dosing and sampling times differed in each study, but the results were always positive (Table 5). The results from treated groups were statistically compared to the negative control results and those with p values of <0.05 were considered positive.

Table 6 shows the results of the seven articles that studied the genotoxic effect of AgNPs through the in vivo comet assay. Only Asare et al. [99] followed OECD TG 489 [66]. Different strains of mouse, rat and rabbit were used as experimental models, and comets were analyzed in cells from liver, lung, testis, blood or bone marrow. AgNPs were administered through the i.v. route (single or 3 days) in four studies and through the p.o. route (single, 5, 35 or 45 days) in three studies. Uncoated and PVP-, silicon-, citrate- or PDDAC-coated AgNPs arranging in size from 5−200 nm were administered to the animals. The doses were higher for oral treatments (5−100 mg/kg) than for intravenous treatments (0.5−25 mg/kg). 

According to OECD TG 489 [66] for the in vivo comet assay, a result is considered positive if at least one of the test doses exhibits a statistically significant increase compared to the concurrent negative control, the increase is related to dose when evaluated with an appropriate trend test and any of the results fall outside the distribution of the historical negative control data for a given species, vehicle, route, tissue, and number of administrations. Asare et al. [99] applied the OECD TG 489 [66] criteria, results from treated groups in the other studies were statistically compared to negative control groups, and those with p values of <0.05 were considered positive. As shown in Table 6, most of the results were negative.

The biodistribution of AgNPs was studied in only three of the articles retrieved: Wen et al., Li et al. and Martins et al. [41,100,107]. In these studies, total Ag was determined through inductively coupled plasma mass spectrometry, without differentiating between Ag ions and AgNPs. Li et al. [100] administered a single dose of 15−100 nm PVP-AgNPs or 10−80 nm silicon-AgNPs and detected Ag in peripheral blood and bone marrow tissue, with a much higher concentration in the bone marrow of animals treated with PVP-AgNPs. After three days of treatment, Ag was also found in the liver. Surprisingly, Li et al. [100] reported negative results in the in vivo MN assay when analyzing blood samples (Table 4). When they performed the in vivo comet assay with liver samples, they obtained negative results in the standard version but positive results in the OGG-1- and Endo-III-modified comet assays (Table 6). Wen et al. [41],intravenous administered a single dose of AgNPs (6.3−629 nm) dose and detected an accumulation of Ag in several tissues, in the following order (highest to lowest): lung > spleen > liver > kidney > thymus > heart. In bone marrow samples from AgNPs- and Ag+-treated animals, the authors observed an increase in both MN and CA with respect to control animals, but the differences were statistically significant only for CA (Table 4 and Table 5). Martins et al. [107] orally administered 90 nm AgNPs for 45 days and observed the higher concentration of AgNPs in blood, followed by liver, and a much lower concentration in kidneys; however, they obtained negative results in the standard in vivo comet assay in both blood and liver (Table 5).

Considering all of the in vivo results, there is no clear evidence that the genotoxic effect of AgNPs is influenced by NPs size or coating; moreover, this aspect was not the specific objective of the in vivo studies analyzed. Only Nallanthighal et al. [102] observed that citrate-AgNPs exerted a greater genotoxic effect than PVP-AgNPs after seven days of oral administration (Table 4).

## 5. General Discussion

When the results of the in vivo and in vitro assays are considered as a whole, it is evident that positive results were obtained for every damage level (i.e., primary DNA damage, gene mutations and CA), although it is important to note that positive results were predominant in the in vitro assays and negative results in the in vivo assays. Even if the in vitro comet results that detected reparable DNA damage are excluded and the mutations detected by the MLA and the in vitro MN assays are included the results are clearly positive. The few exceptions to this rule were obtained with cell lines that are not normally used in the in vitro MN assay (HBEC, MCF-10A, MCF-7, MDA-MB-231 and BALB/3T3A31-1-1). The in vivo results as a whole show that MN and CA assays obtained more positive results than the comet assays. Thus, AgNPs also had the capacity to induce chromosome aberrations in vivo. By contrast, DNA damage was not generally detected in bone marrow, liver, lung or testis with the standard comet assay, but the enzyme-modified versions, which detect mainly oxidized bases, produced more positive results. Moreover, a number of factors such as AgNPs biodistribution (see below) and the experimental in vivo design may account for these results. 

None of the articles selected carried out a comprehensive evaluation of AgNPs genotoxicity through a complete battery of assays that included both in vitro and in vivo experimental designs, as recommended in the ICH and EFSA strategies. Only Wang et al. [84] applied a strategy that included both in vitro and in vivo assays; they evaluated AgNPs genotoxicity through the in vitro MN and comet tests and the in vivo bone narrow MN test, and obtained positive results in all of them. Guo et al. [72] studied the genotoxicity of AgNPs by applying of a core battery of in vitro genotoxicity assays recommended for FDA regulated products: the Ames test, the MLA and the in vitro MN assay. They obtained positive results in all except the Ames test, which produced inconclusive results, thereby confirming that this assay is not suitable for NPs in general, especially for AgNPs.

Biodistribution analysis is essential to understand the behavior of NPs in organisms and to select the tissue to be analyzed for genotoxicity. In general, AgNPs accumulate primarily in the mononuclear phagocyte system (MPS) such as liver and spleen after both oral [108] and intravenous administration [42]. AgNPs accumulation in liver is due to thiol-silver complexes, as silver tends to bind sulfur [108]. Van der Zande et al. [108] showed that after 28 days of oral administration of AgNPs, low concentrations of AgNPs passed through the intestines and AgNPs were eliminated from most organs, except for the brain and testis, eight weeks after treatment. AgNPs biodistribution was not studied in all in vivo genotoxicity assays retrieved in this review, but when the test was performed, the results were consistent with this general behavior, even if the dose, administration route and treatment schedule differed. As mentioned before, Ag has been detected in bone marrow, blood and liver [41,100,107], which are the most commonly used tissues in genotoxicity assays.

AgNPs attach to the cell membrane where they alter permeability and penetrate the cell, thus releasing Ag+ ions. Once inside the cell, Ag+ ions cause damage to structures and biomolecules, alterations in respiration, oxidative stress by radical oxygen species (ROS) and modulation of signal transduction pathways [109,110] The principal mechanism through which NPs cause cell damage, including DNA damage, is based on the exogenous generation of ROS such as superoxide anion, hydroxyl radical, singlet oxygen, hypochlorous acid and hydrogen peroxide which can damage DNA and lead to mutagenesis processes [24,83,103,111,112].AgNPs can also interact with mitochondria and disrupt the electron transport chain, thereby causing ROS production and interrupting of ATP synthesis; ROS increase and a shortage of ATP result in protein and DNA damage [112,113]. 

In addition, AgNPs have been shown to disrupt the DNA repair cell systems, thus leading to depletion of antioxidant molecules and damaging the cell membrane [112]. Furthermore, interaction between AgNPs and DNA can cause DNA shearing or denaturation and disrupt in cell division [109].

The protein function is specific to one conformation and an alteration can cause important cell injuries [114]. Both AgNPs and Ag + ions have been shown to interact with proteins, thereby leading to altered conformation, deactivation and even degradation [112,115,116,117]. When AgNPs bind to proteins, they form corona structures and can express genes involved in DNA damage [112]. 

Ag+ ions released from the AgNPs are bioactive; they have a specific capacity to form complexes with proteins by thiol groups [32,116]. Ag + ions in complexes with proteins can interfere with Na+/Cl- transport and contribute to ROS production, disrupt the physiological activity of proteins and even lead to cell death [32,110,116]. Whether the effects observed in AgNPs testing are produced by the NPs themselves or by the ions that are released is not clear and requires further study [32,116].

By contrast, the results of both in vitro and in vivo comet assays, especially the in vivo assays, do not clearly indicate that the genotoxic effect is produced by oxidative damage. According to Klain et al. [117], the interactions of AgNPs with the SH groups at the active site of Fpg, can reduce the enzyme capacity to detect damage. On the other hand, Magdolenova et al. [118] claimed that in the actual comet assay, Fpg and that NPs, therefore do not affect Fpg activity. The positive Fpg comet results confirm this (Table 3). For all 20 nm and most 200 nm AgNPs tested [99,103] the in vivo comet assay was negative, both standard and enzyme-modified versions (Table 5). Moreover, the mouse strain lacking OGG-1 (OGG1 −/− knockout (KO) mouse) [99], which is supposedly to be more sensitive to oxidative damage, the results of the standard and Fpg-modified comet assays were negative. It is worth noting only a few papers contained results obtained with the comet assay, so more studies are required to shed light on the genotoxic mechanism of action of AgNPs. 

An analysis of the tables suggests that AgNPs size does not influence the genotoxicity of AgNPs, however, this may be due to the way in which doses are expressed. When doses are measured in mg/mL or mg/kg, the surface area of the particles is not considered. AgNPs activity is better correlates with surface area concentration than with mass concentration [119]. Guo et al. [72] observed that when results are corrected by surface area, the differences decrease; if the particles are larger, the same dose contains fewer of them and therefore, has a lower surface area compared to smaller particles. The effect would be smaller [72]. Mass concentration expressed in mg/mL can be converted to surface area concentration expressed in mm^2^/mL using the size and particle number [72]. 

Some authors claim that the effect of AgNPs is influenced by their coating [32,72]. Citrate-coated AgNPs have an increased negative charge which results in high stability and decreases aggregation [120]. Theoretically, the small size and active surface of citrate-coated AgNPs allow them to penetrate the mitochondria and nuclear pore and to create free radicals [101]. With regard to in vivo MN test, silicon coating produced negative results in Li et al. [100] and PVP-coating produced negative results in almost every evaluation [32,100] except for Wang et al. [84]. Moreover, Nallanthighal et al. [32] obtained positive MN results after seven days of oral treatment with citrate coated AgNPs in C57BL/6 mice, but negative results after treatment with PVP-AgNPs under the same conditions. Regarding the in vivo comet assay, silicon- and PVP- coatings produced negative results in the standard comet assay, although Li et al. [100] obtained positive results in Endo-III and OGG-1 modified comet assays in. 

As mentioned above, a physicochemical characterization of nanomaterials is essential to understand their behavior. In the analyzed papers, the main parameters determined were particle size and size diameter, hydrodynamic diameter, polydispersity index, zeta potential, particle morphology and agglomeration state. Dynamic light scattering (DLS) was one of the most widely used techniques to determine particle size, size distribution, zeta potential, hydrodynamic diameter and polydispersity index. Transmission electronic microscopy (TEM) was used to determine particle size, morphology, particle shape and agglomeration. Zeta potential was determined mostly with a Zetasizer instrument. Other techniques such as inductively coupled plasma mass spectrometry (ICP-MS) and X-ray photoelectron spectroscopy (XPS) were applied to determine AgNPs concentrations and elemental composition respectively. 

In summary, looking at all the results together, it appears to indicate that the AgNPs can produce genotoxic effects. Positive results were obtained in all type of assays, both in vitro and in vivo, although characteristics of each AgNPs and test conditions should be considered case-by-case AgNPs, as previously mentioned in the introduction, are highly used in consumer products, medicine and other sectors and this poses a worrying scenery that needs complete risk assessment evaluating real human exposure. 

## Figures and Tables

**Table 1:** In vitro mouse lymphoma assay results.

NP Size (nm)	Coating	Cells	H	µg/mL	Result	Ref.
20	Citrate	L5178Y	4	1–60	+	[72]
	PVP				+	
50	Citrate				+	
	PVP				+	
100	Citrate				+	
	PVP				+	

PVP: polyvinylpyrrolidone; H: treatment duration in hours; μg/mL: range of doses tested; +: positive result, according to OECD TG 490 criteria (see text).

**Table 2:** In vitro micronucleus assay results.

NP Size (nm)	Coating	Cells	H	μg/mL	Result	Ref.
5		HBEC	48	1–20	-	[78]
		TK6	28	1–1.5	+	[80]
10		CHO-K1	24	0.025–2.5	+	[81]
		CHO-XRS5			-	
		JURKAT E61	24	1–25	+	[77]
		TPH-1			+	
15		MCF-10A	24	5.9–23.5	-	[52]
		MCF-7	48	4.1-16.3	-	
		MDA-MB-231		1.2–4.9	-	
		CHO-K1	24	1–5–10	+	[82]
20–50		HepG2	48	12.5–200	+	[83]
		A549			+	
		HepG2	24	12.5–200	+	[79]
		A549			+	
20		JURKAT	24	1–25	+	[77]
		TPH-1			+	
		HepG2 (s)	48	5–15	-	[73]
		CACO2(s)			-	
		HepG2 (a)		1–10	+	
		CACO2 (a)			-	
		HepG2	24	20–160	+	[84]
	PVP				+	
	Citrate	Lymphocytes	44	0.2–25	+	[74]
	bPEI				+	
	Citrate	JURKAT	24	0.1–25	+	
	bPEI				+	
	Citrate	WIL2-NS			+	
	bPEI				+	
	Citrate	L5178Y	4	1.25–4	+	[72]
		TK6		2.5–15	+	
	PVP	L5178Y		1.25–8	+	
		TK6		2.5–30	+	
30	Citrate	HaCat	24	10–40	+	[31]
			48		+	
45		MCF-10A	24	5.9–23.5	-	[52]
		MCF-7	48	4.1–16.3	-	
		MDA-MB-231		1.2–4.9	-	
50		HBEC	48	1–20	-	[78]
		HepG2	4	10–100	-	[75]
			24	2.5–25	-	
		CACO2	4	10–100	-	
			24	2.5–25	-	
		HepG2	40–44	1–20	+	[76]
		CACO2			-	
		JURKAT	24	1–50	+	[77]
		TPH-1			+	
	Citrate	L5178Y	4	1.25–20	+	[72]
		TK6		2.5–120	-	
	PVP	L5178Y		1.25–30	+	
		TK6		2.5–140	-	
90		Balb3T3 A31-1-1	24	0.17–10.60	+	[85]
100		CHO-K1	24	0.025–2.5	-	[81]
		CHO-XRS5			+	
		JURKAT	24	1–50	+	[77]
		TPH-1			+	
	Citrate	L5178Y	4	1.25–35	+	[72]
		TK6		2.5–400	+	
	PVP	L5178Y		1.25–50	+	
		TK6		2.5–400	+	

Size column, samples of NPs of various sizes are expressed as minimum-maximum size; coating column: blank space means no coating, bPEI: branched polyetherimide, PVP: polyvinylpyrrolidone; H: treatment duration in hours; μg/mL: range of doses tested; -: negative result, +: positive result, (some authors followed OECD TG 487, see text for explanation).

**Table 3:** In vitro comet assay results.

NP Size (nm)	Coating	Cells	H	l	SC	Fpg	Endo-III	OGG-1	Ref.
5		HBEC	48	1–20	+				[78]
	PEI + PVP	HepG2	24	0.1–1.6	+	+	-		[87]
		HL-60			+	+	+		
		NHDF			+	+	+		
		HPF			+	+	+		
5–15	PVA	Blood cells	4	1–50 (μM *)	+				[88]
		HepG2			+				
5–20	AOT	HepG2	24	1–10	+				[89]
	CTAB				+				
	PVP				+				
	BSA				+				
	PLL				+				
10–30		MCF-7	24	5–150	+				[90]
10		CACO2	24	1–50	-	+			[53]
		CHO-K1	24	0.025–2.5	+				[81]
		CHO-XRS5			+				
		JURKAT E6-1	24	2.5–20	+				[77]
		TPH-1		1–25	+				
	PVP	BEAS-2B	4	10	-				[91]
			24		+				
	Citrate		4		-				
			24		+				
13–60	Citrate	PK15	24	1–75	+				[92]
			48		+				
15		MCF-10A	24	5.9–23.5	+	-			[52]
		MCF-7		4.1–16.3	+	-			
		MDA-MB-231		1.2–4.9	-	-			
20–50		HepG2	48	12.5–200	+				[83]
		A549			+				
		HepG2	24	12.5–200	+				
		A549			+				
20		JURKAT E6-1	24	5–25	+				[77]
		TPH-1		5–40	+				
		HepG2		20–160	+				[84]
	PVP				+				
27		NIH3T3	6	30.1–90.1	+				[93]
		SVK14		25.4–76.1	-				
30	Citrate	HaCat	24	10–40	+				[31]
			48		+				
35	PVP	Hela	12	1.25–10	+				[94]
			24		+				
		MDA-MB-231	12		+				
			24		+				
		MCF-7	12		+				
			24		+				
		HMEC	24 h	2.5–5	+				[95]
		ECFC			+				
		BMDC	12	0.03–1	+				[96]
40		HepG2	24	0.1–6.7	+	+	+		[87]
		HL-60			+	+	+		
		NHDF			+	+	+		
		HPF			+	-	-		
	Citrate	BEAS-2B	4	10	-				[91]
			24		+				
45		MCF-10A	24	5.9–23.5	-	-			[52]
		MCF-7			-	-			
		MDA-MB-231		2–8.1	-	-			
50		HBEC	48	1–20	+				[78]
		JURKAT E6-1	24	10–50	-				[77]
		TPH-1			-				
		BEAS-2B	4	10	-				[91]
			24		+				
56.4		A549	24	10–50	+				[97]
			48		+				
60		HEK293T	24	10–40	+				[82]
60–105		A549	24	25	-				[97]
75	Citrate	BEAS-2B	4	10	-				[91]
			24		+				
100		CHO-K1	24	0.025–2.5	+				[81]
		CHO-XRS5			+				
	PVP	GMO7492	24	0.01–10	+			+	[98]
		JURKAT E6-1	24	10–50	-				[77]
		TPH-1			-				
105		NIH3T3	6	1.3	+				[99]
		SVK14		2.1	+				
		BJ		2.2	+				
131.5		NIH3T3	6	1.4	+				
		SVK14		2.2	+				
		BJ		2.3	+				

Size column, samples of NPs of various sizes are expressed as minimum-maximum size; coating column, blank space means no coating, AOT: sodium bis(2-ethylhexyl)-sulfosuccinate, CTAB: cetyltrimethylammonium bromide, BSA: bovine serum albumin, PLL: poly-L-lysine, bPEI: branched polyetherimide, PVP: polyvinylpyrrolidone; H: treatment duration in hours, nd: no data available; * μM: treatment concentration micromolar, * µg: treatment expressed in quantity; SC: standard comet assay, Fpg: formamidopyrimidine DNA glycosylase modified comet, Endo-III: endonuclease III modified comet, OGG-1: oxoguanine glycosylase 1 modified comet; +: positive result, -: negative result. Results were statistically evaluated by comparing treated cells with untreated control cells; those with p < 0.05 at any of the concentrations tested were considered as positive.

**Table 4:** In vivo micronucleus assay results.

NPs Size (nm)	Coating	Animal Model	Tissue	Route	Duration	Dose (mg/kg Body Weight)	ST	Result	Ref.
5	PVP	B6C3F1 mouse	Blood	i.v.	Single dose	0.5–20	2 d	-	[100]
10	-	Sprague Dawley rat	Bone marrow	p.o.	5 day	5–100	1 d	+	[101]
20	Citrate	OGG1 −/− KO C57BL/6 mouse	Blood	p.o.	3 day	4	1 d	+	[102]
					7 day			+	
		C57BL/6 mouse			3 day			+	
					7 day			+	
	Citrate	C57BL/6 mouse	Blood	p.o.	7 day	4	1 d	+	[32]
							1 w	+	
							2 w	+	
	PVP						1 d	-	
							1 w	-	
							2 w	-	
		Wistar rat	Bone marrow	i.v.	Single dose	5	1 d	+	[103]
							1 w	+	
							4 w	-	
						10	1 d	+	
							1 w	+	
							4 w	+	
		ICR mouse	Bone marrow	p.o.	28 day	10–250	1 d	+	[84]
	PVP							+	
10-80	Silicon	B6C3F1 mouse	Liver	i.v	Single dose	25	2d	-	[100]
					3 day			-	
200		Wistar rat	Bone marrow	i.v.	Single dose	5	1d	+	[103]
							1w	+	
							4w	-	
6.3-629		Sprague Dawley rat	Bone marrow	i.v.	Single dose	5	1d	-	[41]
15-100	PVP	B6C3F1 mouse	Liver	i.v.	Single dose	25	2d	-	[100]
								-	

Size column, samples of NPs of various sizes are expressed as minimum-maximum size; coating column, blank space means no AgNPs coating, PVP: polyvinylpyrrolidone; OGG1 −/− KO: 8-oxoguanine glycosylase knockout (mouse strain lacking OGG-1); p.o.: oral once a day, i.v.: intravenous, ST: sampling time; d: day post treatment, w: week post treatment; +: positive result, -: negative result. Results from treated groups were statistically compared to negative control results and those with p<0.05 at any of the doses tested were considered as positive.

**Table 5:** In vivo chromosome aberration assay results.

Size (nm)	Coating	Animal Model	Tissue	Route	Duration	Dose (mg/kg Body Weight)	ST	Result	Ref.
10		Sprague Dawley rat	Bone marrow	p.o.	5 day	5–100	1 d	+	[101]
		Albino rat	Bone marrow	i.p.	28 day	1–4	0 d	+	[104]
6–629		Sprague Dawley rat	Bone marrow	i.v.	Single dose	5	1 d	+	[41]

Size column: samples of NPs of various sizes are expressed as minimum-maximum size; coating column: blank space means no coating; p.o.: oral once a day, i.v.: intravenous, i.p.: intraperitoneal, ST: sampling time; 0d: day of finishing treatment, d: day post treatment; +: positive result, -: negative result. Results from treated groups were statistically compared to negative control results and those with p < 0.05 at any of the doses tested were considered as positive.

**Table 6:** In vivo comet assay results.

NPs Size (nm)	Coating	Animal Model	Tissue	Route	Duration	Dose (mg/kg Body Weight)	ST	SC	Fpg	Endo-III	OGG-1	Ref.
5		Swiss albino mouse	Liver	p.o.	Single dose	5–100	3 h	+				[105]
							1 d	+				
				p.o.w.	35 day	10–20	3 h	+				
8	Citrate	New Zeland white rabbit	Liver	i.v.	Single dose	0.5–5	7 d	+				[106]
							28 d	+				
10		Sprague Dawley rat	Bone marrow	p.o.	5 day	5–100	0	+				[101]
10-80	Silicon	B6C3F1 mouse	Liver	i.v.	3 day	25	3 h	-		+	+	[100]
20		Wistar rat	Bone marrow	i.v.	Single dose	5	1 d	-				[103]
							1 w	-				
							4 w	-				
						10	1 d	-				
							1 w	-				
							4 w	-				
		OGG1 −/− KO C57BL/6 mouse	Lung	i.v.	Single dose	5	1 d	-	-			[99]
							1 w	-	-			
			Liver				1 d	-	-			
							1 w	-	-			
			Testis				1 d	-	-			
							1 w	-	-			
		C57BL/6 mouse	Lung				1 d	-	-			
							1 w	-	-			
			Liver				1 d	-	-			
							1 w	-	-			
			Testis				1 d	-	-			
							1 w	-	-			
90		Wistar rat	Blood	p.o.	45 day	0.5	0	-				[107]
			Liver				0	-				
15–100	PVP	B6C3F1 mouse	Liver	i.v.	3 day	25	3 h	-		+	+	[100]
200		Wistar rat	Bone marrow	i.v.	Single dose	5	1 d	-				[106]
							1 w	-				
							4 w	-				
		OGG1 −/− KO C57BL/6 mouse	Lung	i.v.	Single dose	5	1 d	-	-			[101]
							1 w	+	-			
			Liver				1 d	-	-			
							1 w	-	-			
			Testis				1 d	-	-			
							1 w	-	-			
		C57BL/6 mouse	Lung				1 d	-	-			
							1 w	-	+			
			Liver				1 d	-	-			
							1 w	-	-			
			Testis				1 d	-	-			
							1 w	-	+			

Size column, sets of NPs of various sizes are expressed as minimum-maximum size; coating column: blank space means no AgNPs coating, PVP: polyvinylpyrrolidone; OGG-1 −/− KO: 8-oxoguanine glycosylase knockout (mouse strain lacking OGG-1); p.o.: oral once a day, i.v.: intravenous, ST: sampling time; 0: day of finishing treatment, h: hour post treatment, d: day post treatment, dt: day of treatment, w: week post treatment; SC: standard comet assay; Fpg: formamidopyrimidine DNA glycosylase modified comet; Endo-III: endonuclease III modified comet; OGG-1: oxoguanine glycosylase 1 modified comet; +: positive result; -: negative result.. Asare et al. [99] applied the OECD TG 489 criteria (see text for explanation). In other studies, results from treated groups were statistically compared to negative control results and those with p < 0.05 at any of the doses tested were considered as positive.
